# Prediction of the curing time to achieve maturity of the nano-cement based concrete using the Weibull distribution model: A complementary data set

**DOI:** 10.1016/j.dib.2015.05.018

**Published:** 2015-06-12

**Authors:** Byung Wan Jo, Sumit Chakraborty, Heon Kim

**Affiliations:** Department of Civil and Environmental Engineering, Hanyang University, Seoul 133791, South Korea

**Keywords:** Nano-cement, Ordinary Portland cement, Weibull distribution, Curing time, Maturity of concrete

## Abstract

This data article provides a comparison data for nano-cement based concrete (NCC) and ordinary Portland cement based concrete (OPCC). Concrete samples (OPCC) were fabricated using ten different mix design and their characterization data is provided here. Optimization of curing time using the Weibull distribution model was done by analyzing the rate of change of compressive strength of the OPCC. Initially, the compressive strength of the OPCC samples was measured after completion of four desired curing times. Thereafter, the required curing time to achieve a particular rate of change of the compressive strength has been predicted utilizing the equation derived from the variation of the rate of change of compressive strength with the curing time, prior to the optimization of the curing time (at the 99.99% confidence level) using the Weibull distribution model. This data article complements the research article entitled “Prediction of the curing time to achieve maturity of the nano-cement based concrete using the Weibull distribution model” [1].

## 1 Specifications table

**Table:** 

Subject area	Materials science
More specific subject area	Cement based material (Nano-cement and ordinary Portland cement)
Type of data	Table and graph
How data was acquired	The data were acquired using a universal testing machine (Schimadzu, CCM-200A; Shimadzu Corporation, Japan).
Data format	Raw and processed
Experimental factors	The compressive strength of the concrete fabricated in this investigation was estimated in accordance with the Korean standard KS F 2405 [2]. The compressive strength of cylindrical concrete sample of the dimension 10 cm×20 cm was measured using a universal testing machine with a loading rate 0.06 MPa/min. To obtain an average compressive strength, six concrete samples from each batch of the concrete mix design were tested. During the test, maximum load obtained at the complete failure of the specimen was estimated.
Experimental features	In this investigation, concrete samples were fabricated using ordinary Portland cement using ten different mix design, followed by compressive strength measurement at four different curing times. Subsequently, the required curing time to achieve a particular rate of change of the compressive strength was predicted utilizing the equation derived from the variation of the rate of change of compressive strength with the curing time, prior to the optimization of the curing time (at the 99.99% confidence level) using the Weibull distribution model.
Data source location	Department of Civil Engineering, Hanyang University, Seoul, South Korea
Data accessibility	The data are supplied with this paper

## 2 Value of the data

• Evaluation of the compressive strength of ordinary Portland cement compared to nano-cement based concrete.
• Estimation of the rate of change compressive strength of ordinary Portland cement as a function of curing time.
• Optimization of the curing time to achieve a particular rate of change of compressive strength of OPCC using the Weibull distribution model.
• Weibull distribution analysis of the curing time to achieve maturity of the OPCC.

## 3 Data, experimental design, materials and methods

Strength data presented here are from ten different concrete samples (OPCC) fabricated to compare nano-cement based concrete (NCC) with ordinary Portland cement based concrete (OPCC).

## 4 Sample preparation method

In this investigation, concrete samples were fabricated using ordinary Portland cement, variable amounts fine and coarse aggregate and water (Table 1). Initially, required amount of cement was mixed with the required amount of fine and coarse aggregate, followed by mixing with quantified amount of water. Thereafter, the concrete samples were cast immediately into the mold of the dimension 10 cm×20 cm. After complete setting, the samples were removed from the mold and allowed to cure for four different curing times such as 3, 7, 14 and 28 days.

## 5 Characterization and data analysis

**Fig. 1:**
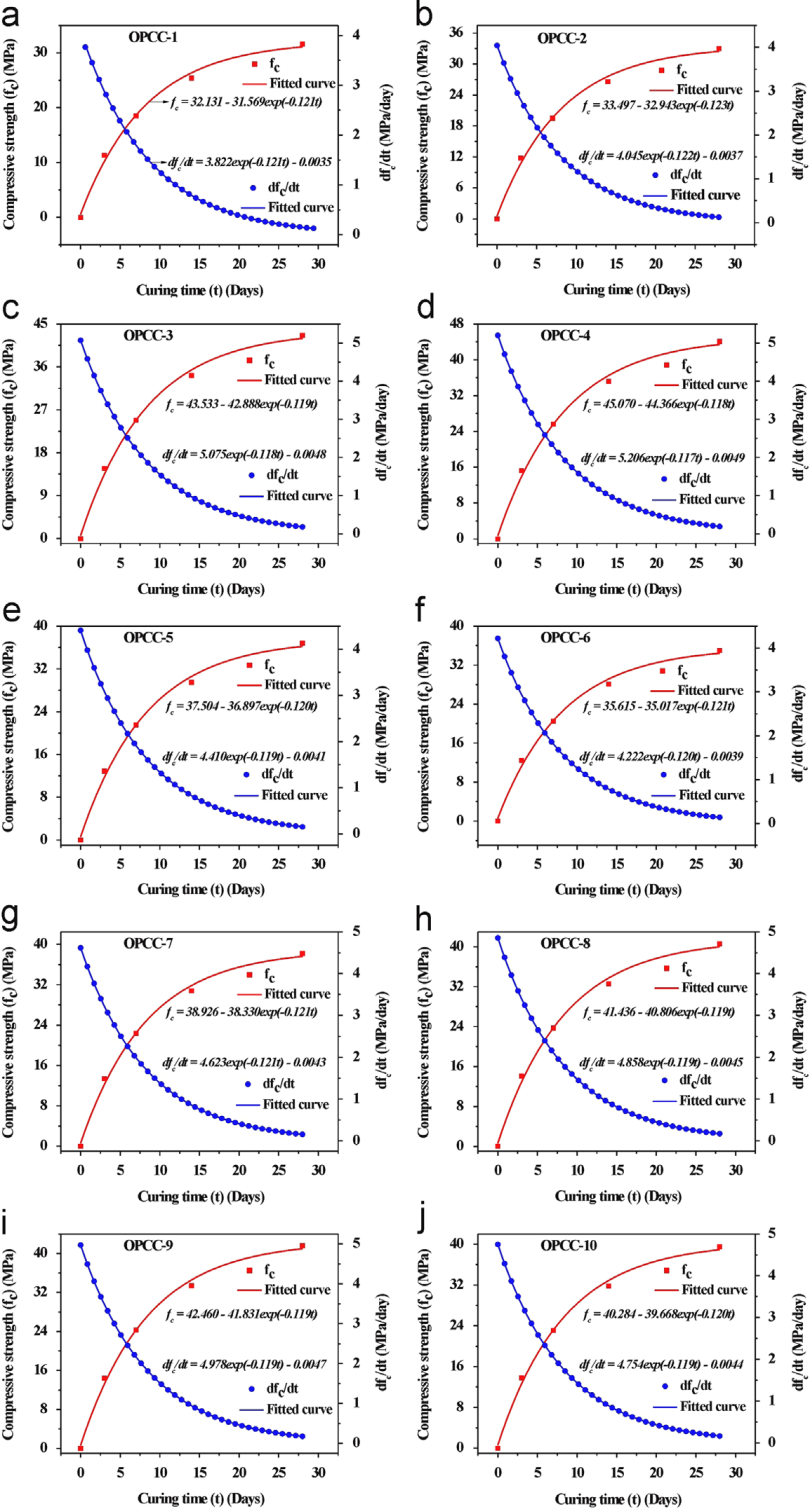
Variation of the compressive strength (*f*_*c*_) of ordinary Portland cement based concrete as a function of the curing time (*t*), fitting of the compressive strength data with an exponential equation to evaluate the trend for the increment of the compressive strength with curing time as well as the variation of the rate of change of the compressive strength (*df*_*c*_/*dt*) of the ordinary Portland cement based concrete as a function of the curing time (*t*) and the fitting of the rate of change of compressive strength with an exponential equation to predict the trend for the decrement of the rate of change of the compressive strength with curing time, (a) OPCC-1, (b) OPCC-2, (c) OPCC-3, (d) OPCC-4, (e) OPCC-5, (f) OPCC-6, (g) OPCC-7, (h) OPCC-8, (i) OPCC-9, (j) OPCC-10.

The prime focus of this investigation was to optimize the curing time using Weibull distribution model. Initially, the compressive strength of concrete (OPCC) was measured using a universal testing machine with a loading rate 0.06 MPa/min in accordance with the Korean standard KS F 2405 [2]. The compressive strengths of ten different mix design of the ordinary Portland cement based concrete (OPCC) are presented in Table 2. The plot of compressive strength vs curing time as well as the plot of the rate of change of compressive strength (d*f*_*c*_/d*t*) vs curing time of the ten different mix design of OPCC is presented in Fig. 1. A trend line for the variation of compressive strength with curing time was predicted. Thereafter, a first order derivative of the data points of trend line w.r.t. curing time was calculated to obtain a rate of change of compressive strength. Additionally, a best fitted equation of the plot of the rate of change of compressive strength (*df*_*c*_/*dt*) vs curing time of each type of concrete was estimated. The values of the various parameters of the best fitted equation are tabulated in Table 3. From this best fitted equation of the each type of concrete mix design, the times (*t*_*r*1_, *t*_*r*2_, *t*_*r*3_, and *t*_*r*4_) required to achieve a different rate of change of compressive strength ((*df*_*c*_/*dt*)=(*df*_*c*_/*dt*)_max_×10^−2^, (*df*_*c*_/*dt*)_max_×10^−3^, (*df*_*c*_/*dt*)_max_×10^−4^, and (*df*_*c*_/*dt*)_max_=0) were estimated (Table 4). Analyzing the results, a range of the curing time is observed to achieve a particular rate of change of compressive strength of the ordinary Portland cement based concrete fabricated using ten different mix design. Therefore, to normalize this range of the data, a widely used statistical model (Weibull distribution) has been selected. Using this two parameter Weibull distribution model, we are trying to normalize the data at 99.99% probability.

**Fig. 2:**
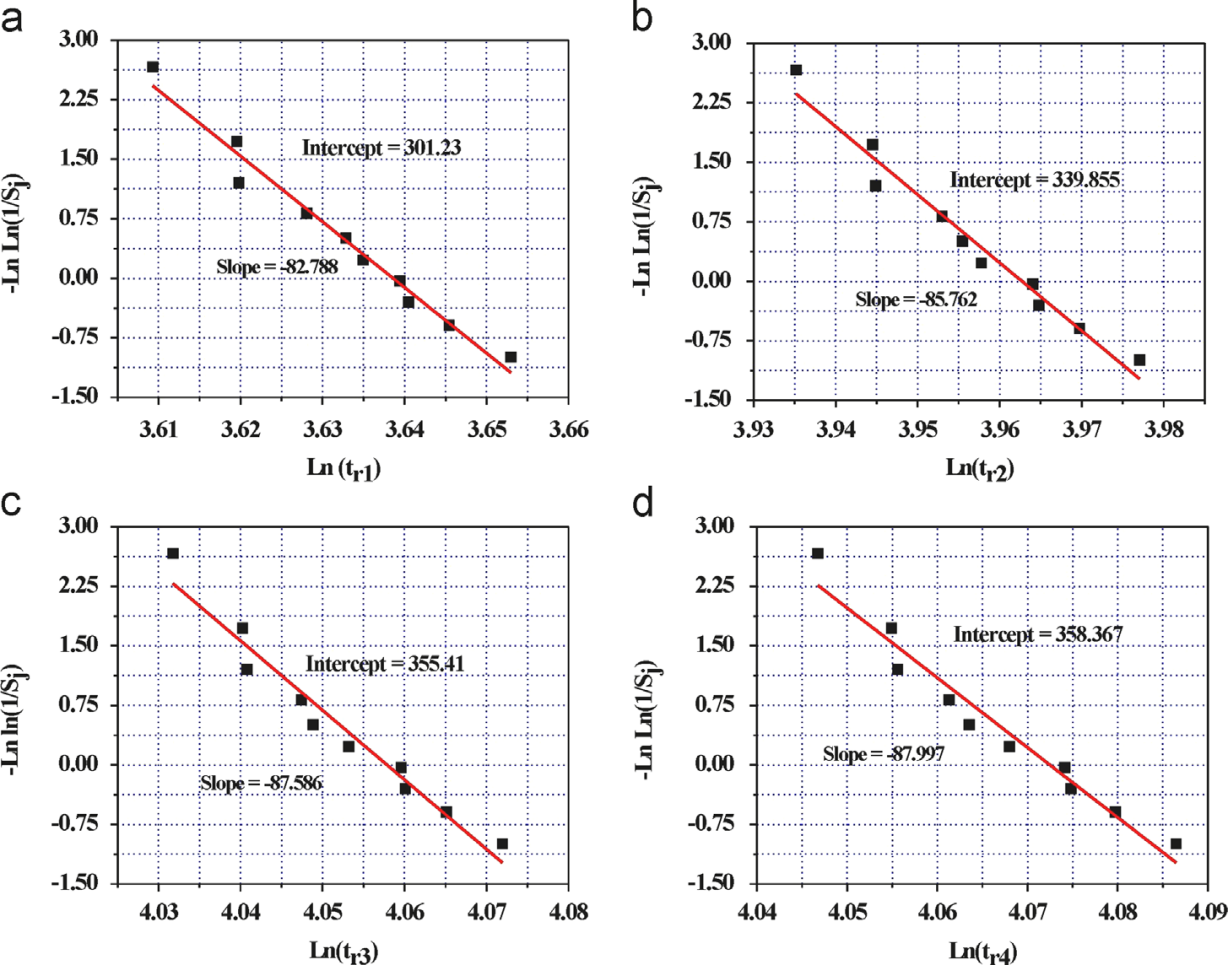
Weibull fitting –LnLn(1/*Sj*) vs Ln(*t*_*r*_) for the ordinary Portland cement based concrete samples to predict the Weibull modulus, *σ*_0_ (37% probability) and *σ* (99.99% probability). (a) –LnLn(1/*Sj*) vs Ln(*t*_*r*1_) (b) –LnLn(1/*Sj*) vs Ln(*t*_*r*2_) (c) –LnLn(1/*Sj*) vs Ln(*t*_*r*3_) and (d) –LnLn(1/*Sj*) vs Ln(*t*_*r*4_).

The probability function of two-parameter semi-empirical distribution (Weibull distribution) is given by Barsoum [3]. Hence, to analyze the curing time such as *t*_*r*1_, *t*_*r*2_, *t*_*r*3_, and *t*_*r*4_ of the OPCC using the Weibull distribution model, initially survival probability (*S*) was calculated. Determination of the survival probability (*S*) for each set of the data, such as *t*_*r*1_, *t*_*r*2_, *t*_*r*3_, and *t*_*r*4_ leads to predict the *m* and *σ*_0_ value. From this *m* and *σ*_0_, the design value (*σ*) of the curing time was calculated. Where *m* is a shape factor usually referred as Weibull modulus [3], *σ* is the design value of the curing time (at the survival probability equal to 99.99%) to achieve a particular rate of change of the compressive strength and *σ*_0_ is a normalizing parameter (at the survival probability equals to 1/*e*, i.e. 37%). In this study, *σ* refers to a minimum value of the *t*_*r*1,_ *t*_*r*2_, *t*_*r*3_, and *t*_*r*4_ at the 99.99% confidence level. It means that a minimum value of *t*_*r*1,_ *t*_*r*2_, *t*_*r*3_, and *t*_*r*4_, which will be achieved in 99.99% case, if it is predicted for 100 times. Accordingly, *σ*_0_ refers to a minimum value of the *t*_*r*1,_ *t*_*r*2_, *t*_*r*3_, and *t*_*r*4_ at the 37% confidence level. It indicates that a minimum value of *t*_*r*1,_ *t*_*r*2_, *t*_*r*3_, and *t*_*r*4_, which will be achieved in 37% case, if it is predicted for 100 times. Table 5 represents the values of *S*_*j*_ and Ln(*t*_*r*1_) of the OPCC. Likewise *t*_*r*1_, the values of Ln(*t*_*r*2_), Ln(*t*_*r*3_) and Ln(*t*_*r*4_) were calculated. The plot of –LnLn(1/*S*_*j*_) vs Ln(*t*_*r*1_), –LnLn(1/*S*_*j*_) vs Ln(*t*_*r*2_), –LnLn(1/*S*_*j*_) vs Ln(*t*_*r*3_) and –LnLn(1/*S*_*j*_) vs Ln(*t*_*r*4_) of OPCC are shown in Fig. 2. From this plot, *σ*_0_ is calculated using the slope (*m*) and intercept values for the OPCC. The values of the Weibull modulus *m*, *σ*_0_ (predicted curing time at survival probability=37%) and *σ* (predicted curing time at survival probability=99.99%) for ordinary Portland cement based concrete (OPCC) are represented in Table 6. From the analysis, the values of *t*_*r*1_, *t*_*r*2_, *t*_*r*3_, and *t*_*r*4_ at the 99.99% confidence level of ordinary Portland cement based concrete are estimated to be 37.3, 51.7, 56.8 and 57.7 days, respectively. Nonetheless, the values of the nano-cement based concrete were calculated to be 19.57, 20.91, 21.05 and 21.07 days, respectively [1]. Therefore, it is assessed that ordinary Portland cement requires more time (58 days) to be cured completely as compared to that of the nano-cement based concrete (21 days). Although, it was reported by ACI Committee 308 [4] that different types of cement take different times to cure completely. Additionally, ACI 214R-02 [5] reported that usually 28 days are required to yield adequate curing of the Portland cement based concrete.

## Figures and Tables

**Table 1:** Formulations code and mixing proportion of the components for the fabrication of ordinary Portland cement based concrete.

**Formulations code**	**Component weight (kg/m**^**3**^**)**			
	**Ordinary Portland cement**	**Water**	**Fine aggregate**	**Coarse aggregate**
OPCC-1	420	400	396	924
OPCC-2	420	400	436	884
OPCC-3	420	400	474	846
OPCC-4	420	400	516	804
OPCC-5	420	400	554	766
OPCC-6	420	380	500	820
OPCC-7	420	400	500	820
OPCC-8	420	420	500	820
OPCC-9	420	440	500	820
OPCC-10	420	460	500	820

**Table 2:** Compressive strength of ordinary Portland cement based concrete cured for different times (days).

**Formulations code**	**Compressive strength (MPa) of the concrete samples cured for different curing time (days)**			
	**3 days cured**	**7 days cured**	**14 days cured**	**28 days cured**
OPCC-1	11.3	18.5	25.4	31.6
OPCC-2	11.8	19.5	26.6	33.0
OPCC-3	14.7	24.8	34.2	42.6
OPCC-4	15.2	25.6	35.2	44.1
OPCC-5	12.9	21.5	29.5	36.8
OPCC-6	12.4	20.5	28.1	35.0
OPCC-7	13.4	22.4	30.8	38.2
OPCC-8	14.1	23.7	32.6	40.6
OPCC-9	14.4	24.3	33.4	41.6
OPCC-10	13.8	23.1	31.8	39.5

**Table 3:** The values of the different parameters of the exponential equation for different concrete samples obtained by fitting of the rate of change of the compressive strength (*df*_*c*_/*dt*) vs time (*t*) plot.

**Sample code**	**Values of the different parameters of the equation*****df***_***c***_***/dt=(df***_***c***_***/dt)***_***28d***_***+(df***_***c***_***/dt)***_***max***_**×*****exp(R***_**0**_**×*****t)*****from*****df***_***c***_**/*****dt*****vs*****t*****plot**			***R***^**2**^
	(*df*_*c*_/*dt*)_28d_	(*df*_*c*_/*dt*)_max_	*R*_0_	
OPCC-1	−0.00354	3.822	−0.1210	0.999
OPCC-2	−0.00371	4.045	−0.1220	0.999
OPCC-3	−0.00476	5.075	−0.1179	0.999
OPCC-4	−0.00491	5.206	−0.1170	0.999
OPCC-5	−0.00410	4.410	−0.1190	0.999
OPCC-6	−0.00392	4.222	−0.1200	0.999
OPCC-7	−0.00429	4.623	−0.1210	0.999
OPCC-8	−0.00454	4.859	−0.1186	0.999
OPCC-9	−0.00466	4.978	−0.1185	0.999
OPCC-10	−0.00443	4.754	−0.1194	0.999

**Table 4:** The estimated time to reach a different value of the (*df*_*c*_/*dt*) for each concrete mix design using the equation represented in Table 3.

**Sample code**	**Time (days) required to reach a different value of the rate of change of compressive strength (*****df***_***c***_**/*****dt*****)**			
	***t***_***r*****1**_^a^	***t***_***r*****2**_^b^	***t***_***r*****3**_^c^	***t***_***r*****4**_^d^

OPC-1	37.33	51.67	56.87	57.72
OPC-2	36.94	51.17	56.36	57.21
OPC-3	38.30	52.97	58.27	59.13
OPC-4	38.59	53.36	58.67	59.53
OPC-5	37.90	52.22	57.25	58.05
OPC-6	37.64	52.09	57.33	58.18
OPC-7	37.32	51.65	56.84	57.68
OPC-8	38.07	52.67	57.95	58.80
OPC-9	38.11	52.71	57.98	58.84
OPC-10	37.82	52.34	57.58	58.44

a Time required to reach the value (*df*_*c*_/*dt*)_max_×10^−2^, where (*df*_*c*_/*dt*)_max_ is the maximum value of the rate of change of compressive strength (*df*_*c*_/*dt*).

b Time required to reach the value (*df*_*c*_/*dt*)_max_×10^−3^.

c Time required to reach the value (*df*_*c*_/*dt*)_max_×10^−4^.

d Time required to reach the value of (*df*_*c*_/*dt*)=0.

**Table 5:** Summary of the calculated data for *t*_*r*1_ of ordinary Portland cement based concrete for the prediction of Weibull modulus and *σ*_0_ values by Weibull fitting.

**Ordinary Portland cement based concrete (OPCC)**				
**Rank**	***t***_***r*****1**_	***S***_***j***_	**−Ln(Ln(1/*****S***_***j***_**))**	**Ln(*****t***_***r*****1**_**)**
1	36.94	0.933	2.663843	3.609
2	37.32	0.837	1.723263	3.620
3	37.33	0.740	1.202023	3.620
4	37.64	0.644	0.821667	3.628
5	37.82	0.548	0.508595	3.633
6	37.90	0.452	0.230365	3.635
7	38.07	0.356	−0.03292	3.639
8	38.11	0.260	−0.29903	3.640
9	38.30	0.163	−0.59398	3.645
10	38.59	0.067	−0.99269	3.653

**Table 6:** The statistically analyzed and optimized value of the curing time required to reach a particular rate of change of the compressive strength at 37% and 99.99% confidence level (probability).

**Components**	**Intercept**^a^	**Slope or Weibull modulus (*****m*****)**^b^	***σ***_**0**_**Values at (1/*****e*****) 37% probability**^c^	***σ*****At 99.99% probability**^d^
*t*_*r*1_ (days)	301.23	−82.788	38.0	37.3
*t*_*r*2_ (days)	339.86	−85.762	52.6	51.7
*t*_*r*3_ (days)	355.41	−87.586	57.8	56.8
*t*_*r*4_ (days)	358.37	−87.997	58.7	57.7

a Intercept.

b Slope for each component have been calculated from the respective figures of Fig. 2.

c *σ*_0_ is the value of the component like *t*_*r*1,_*t*_*r*2,_*t*_*r*3_ and *t*_*r*0_ at 37% confidence level.

d *σ* is the value of the component like *t*_*r*1,_*t*_*r*2,_*t*_*r*3_ and *t*_*r*04_ at 99.99% confidence level.
